# Contactless Heart and Respiration Rates Estimation and Classification of Driver Physiological States Using CW Radar and Temporal Neural Networks

**DOI:** 10.3390/s23239457

**Published:** 2023-11-28

**Authors:** Amal El Abbaoui, David Sodoyer, Fouzia Elbahhar

**Affiliations:** COSYS-LEOST, University Gustave Eiffel, F-59650 Villeneuve d’Ascq, France; david.sodoyer@univ-eiffel.fr

**Keywords:** vital signs, CW radar, heart and respiration rate, physiological state, temporal neural networks, Bi-LSTM, CNN, TCN, CRNN

## Abstract

The measurement and analysis of vital signs are a subject of significant research interest, particularly for monitoring the driver’s physiological state, which is of crucial importance for road safety. Various approaches have been proposed using contact techniques to measure vital signs. However, all of these methods are invasive and cumbersome for the driver. This paper proposes using a non-contact sensor based on continuous wave (CW) radar at 24 GHz to measure vital signs. We associate these measurements with distinct temporal neural networks to analyze the signals to detect and extract heart and respiration rates as well as classify the physiological state of the driver. This approach offers robust performance in estimating the exact values of heart and respiration rates and in classifying the driver’s physiological state. It is non-invasive and requires no physical contact with the driver, making it particularly practical and safe. The results presented in this paper, derived from the use of a 1D Convolutional Neural Network (1D-CNN), a Temporal Convolutional Network (TCN), a Recurrent Neural Network particularly the Bidirectional Long Short-Term Memory (Bi-LSTM), and a Convolutional Recurrent Neural Network (CRNN). Among these, the CRNN emerged as the most effective Deep Learning approach for vital signal analysis.

## 1. Introduction

The contactless monitoring of a person’s vital signs is crucial, especially when it comes to evaluating the condition of the driver. This topic has gained attention from researchers in the field of road safety. Factors such as fatigue, stress, distraction, and other emotional states can have an effect on driving ability and increase the risk of road accidents.

To monitor and estimate the alertness of the driver at any given time, various indicators reflecting their physiological state can be considered. Changes in alertness levels correspond to changes in the driver’s psychophysiological state. Hence, technologies that detect drivers physiological signals have become increasingly important for improving road safety [1,2,3]. The physiological signals used for assessing the functional state of drivers include cerebral activity, cardiac rhythm, muscular tension, and respiratory patterns. This paper will specifically focus on heart and respiratory rates as indicators for evaluating the driver’s state.

Researchers have proposed various approaches based on traditional methods for measuring physiological signals, such as electrocardiography (ECG) [4,5] and spirometry [6]. However, these methods are invasive and require instrumentation in direct contact with the driver, which can be cumbersome and potentially dangerous. Non-invasive solutions have been proposed, such as the use of video cameras, photoplethysmography (PPG) [7,8], or pressure sensors on the seat [9]. Nevertheless, these non-invasive solutions can still be cumbersome and do not allow for accurate measurement of physiological signals.

A promising approach for measuring driver physiological signals involves the use of CW radar for heart rate and respiration measurement [10,11]. This contactless method is non-invasive, making it particularly convenient and safe. CW radars use electromagnetic waves to measure the distance and speed of moving objects, which allows for measuring the distance between the radar and the driver’s chest, which moves in rhythm with respiration and heartbeats. Also, CW Doppler radars consume less power and have a simple hardware architecture. The relative displacement information obtained via the CW Doppler radar can be employed to estimate the heart rate in single-person scenarios [12].

CW radar acquires all small vibrations generated on the chest surface by cardiac and respiration activities. It is susceptible to chest vibrations that are unrelated to heartbeats or breathing, such as body movements, as well as interference from other individuals [12]. Reducing the radar’s susceptibility to the propagation channel and driver body movement constitutes a primary challenge. The amplitude of the heartbeat signal is considerably smaller (between 0.2 and 0.5 mm) compared to the thorax displacement caused by respiration (between 4 and 10 mm). Thus, separating the frequencies of each heart and breathing rhythm emerges as the second challenge for researchers in this field [13,14].

To overcome this problem of harmonies, the authors in [15,16] propose signal processing algorithms based on simple filtering or the heart rate frequency estimation using spectral analysis and to separate the heart rate and respiration signals from radar CW measurements. Authors in [15] used the analysis of temporal variations in the signals in successive time windows for processing via the Fast Fourier transform (FFT) [15] or the Wavelet transform (WT) methods [16]. Various approaches using complex signal processing methods are proposed in [14,17,18,19]: In [14], the author introduces an approach based on cyclostationarity techniques to extract heartbeat and respiration rates from vital signals obtained with a 2.4 GHz CW radar, without being dependent on environmental noise and random body movements. This is achieved via the derivation of order 1 and 2’s cyclostationarity moments and the second cyclic cumulant. Furthermore, in [17], the authors focused on extracting the harmonic signal of heartbeats from the vibrations of the chest surface gathered via a Continuous-Wave Doppler Radar System (CW-DRS) equipped with a band-pass filter. This method assumes that respiration does not occur within the heartbeat’s harmonic region. The method proposed in [18] uses a Doppler radar and Empirical Mode Decomposition (EMD) to filter out the noise and detect respiration signals. This signal is filtered to isolate respiration frequencies before being analyzed using the Short-Time Fourier Transform to determine the breathing rate. However, all of the deterministic approaches mentioned above are complex methods and their results depend on the environment of the application and the predefined conditions, which makes them less flexible and less precise.

The analysis and interpretation of raw radar data can be challenging due to several factors, as mentioned in the previous section, such as environment and harmonies. Machine Learning (ML) techniques have been used as a non-deterministic approach to extract physiological signals from the radar data in order to overcome the limits of deterministic methods. Many state-of-the-art solutions use various types of filters to separate the heart and respiration rates. In [20], the authors propose using the gamma filter to model the time series heartbeat signal, accounting for respiration and respiration artifacts. The approach uses a gamma filter to isolate the heart rate from radar-measured signals, providing an effective and non-invasive method for heart rate monitoring. In [21,22], the authors suggest the use of the Kalman filter to update the band-pass filter limits for parameter estimations while considering heart rate measurement and reducing noise in vital signs. However, given the Kalman filter assumptions, it is required to selectively filter out corrupted data caused by arbitrary user motions in order to prevent subsequent vital sign estimates from being tainted [21].

Other approaches are based on unsupervised or supervised machine-learning algorithms to predict vital parameters from time series [23] or to extract pertinent information, such as arrhythmia detection [24], based on electrocardiography (ECG) signals. Furthermore, CW Doppler radar systems have integrated Deep Learning methodologies, such as detecting heartbeats [25,26]. The first results indicate promising advantages in terms of heartbeat detection latency and source separation capabilities (resistance of heartbeat detection to respiration or random body motions) compared to traditional methods. Deep neural networks can learn to detect physiological signals by analyzing raw radar data, extracting relevant features, and estimating heart and respiration rates. the research in [26] proposes the use of convolutional neural networks (CNN) in order to estimate the heart rate from the measured signals using an ultra-wideband radar (UWB). This approach focuses on person-specific identification, with the CNN being trained separately for each subject, primarily due to the lack of available training data. In [12], the authors propose an artificial neural network (ANN) as the main signal processing element, which is trained to detect heartbeats accurately in real time, but most of the methods mentioned above allow us to extract either the heart rate or the respiratory rate but not both at the same time. Recently, the research in [27] proposes the use of a deep learning framework utilizing a convolutional neural network to estimate the heart rate (Fc) and respiration rate (Fr) in real time using a dataset measured during sleep via a UWB radar with a sampling frequency $f_{e}=23$,328GHz and a window size of 15 s in order to detect artifacts. In addition to resolving this problem, the research uses a Continuous Wavelet Transform (CWT) as a pre-processing method to extract the characteristics of each signal.

This paper proposes two approaches: one for estimation and the other for classification aimed at monitoring the driver’s vital signs and estimating his physiological state. The dataset used in this study was obtained from a CW radar with $f_{e}=100\;\mathrm{Hz}$ and a window size of 50 s, which represents a logical duration to estimate the physiological state of the driver. This presents a difference between our study and [27], which uses a 15 s window. Their focus was not on detecting changes in the physiological state but rather on working exclusively with the sleep state. The approaches presented in this paper employ different Deep Learning models: 1D-CNN; the Recurrent Neural Network (RNN); in particular, the Bi-LSTM network, using its ability to remember long-term dependencies in sequential data; and the TCN, which is adept at handling long sequences with complex patterns. Additionally, our paper introduces the CRNN model, which combines the benefits of CNN and Bi-LSTM to achieve a robust performance in detecting and extracting the heart and breath rate values, while also classifying the different physiological states of the driver based on the temporal vital signs measured via the CW radar.

The rest of this paper is organized as follows. Section 2 introduces and discusses the architectures and characteristics of the four proposed Deep Learning models. Section 3 focuses on the CW radar function and data processing. Section 4 represents the results of our models. Finally, Section 5 concludes the study and provides suggestions for future research.

## 2. Models Proposed

This section presents the different Deep Learning models in order to detect and extract the value of the heart rate and the respiratory rate from the temporal vital signs and also to classify the different states of the driver (fatigue or drowsiness, resting or normal state, and the state of stress). We have tested several Deep Learning models to evaluate each model’s performance and contribution to our problem’s study.

### 2.1. 1D-CNN

The 1D-CNN is a Deep Learning technique that involves applying a series of convolution filters to a one-dimensional sequence of data, such as vital signs in our case.

**Figure 1:**
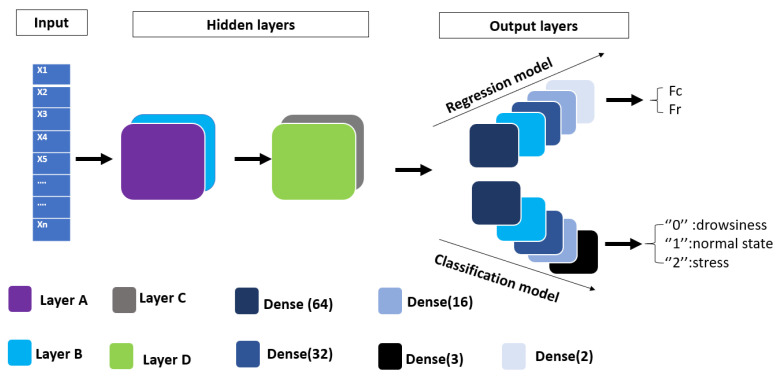
The general architecture of the proposed models.

In our specific approach, we use a 1D-CNN which is comprised of two convolution layers of 128 units with a filter size of 512 (layer A and layer C in Figure 1, as well as a MaxPooling-1D layer (layer B and layer D)) to reduce the dimensionality of the extracted features. These convolution layers allow us to extract relevant features from the input signal. We have used a network of dense layers (fully connected) for the output layers. In our study, we employ CNNs to solve regression and classification problems.The final layer of our model of regression outputs two values, corresponding to the heart rate and the respiratory rate extracted from the time-series signals. The final layer of our classification outputs three values, corresponding to three classes representing the physiological states of the driver as shown in Figure 1.

Figure 1 illustrates the general architecture of the four models we have proposed. We aimed to maintain a similar structure for all four models while modifying the hidden layers section, as explained in Section 2. However, the input section is the same for all four models: each input (xi) corresponds to a vital sign, labeled by the heart and respiratory rates (Fc, Fr) for regression, and by three different physiological states of the driver, namely fatigue or drowsiness, normal state, and stress.

### 2.2. TCN

**Figure 2:**
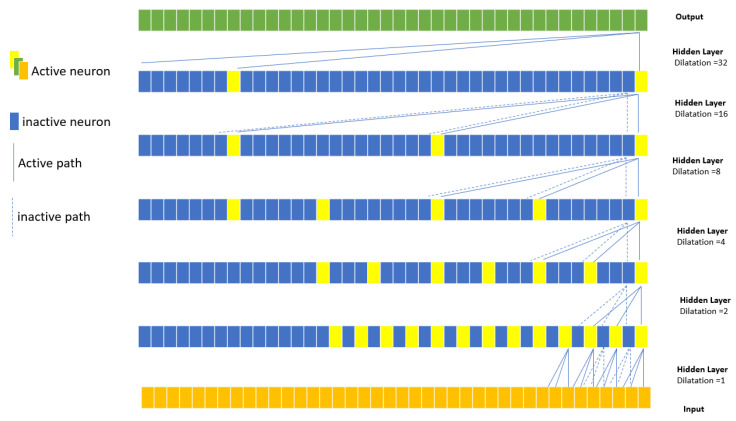
A dilated causal convolution with dilation factors d = 1, 2, 4, 8, 16, and 32 and a filter size k = 3.

In the context of analyzing CW radar signals for the extraction of heart and respiratory frequencies, we have explored an innovative approach using TCN [28]. This is an extension of our previous model that uses 1D CNN, where we have applied a series of convolution filters to a one-dimensional sequence of vital signals. The TCN model proposed consists of a temporal convolution block with dilations ranging from 1 to 32 as hidden layers, as shown in Figure 2. This allows the network to learn dependencies at different time intervals. The convolution layers are complemented by residual connections and causal padding to ensure that the prediction at each instant is based on past and current data. As for the output layers, the model has several fully connected layers (Dense), each followed by a Dropout regularization layer to control overfitting. The last two units of the network are dedicated to predicting heart and respiratory frequencies, which are used for the regression output. For the classification, the TCN output corresponds to the three physiological states of the driver.

This TCN approach offers an improvement over our old 1D CNN-based model, bringing a greater ability to understand temporal dependencies in the signal data and requiring fewer parameters than the CNN. This improvement can lead to a more accurate extraction of heart and respiratory frequencies from raw CW signals.

### 2.3. Bi-LSTM

Bi-LSTM is particularly well suited for detecting vital signals from the CW radar data, due to their ability to process temporal sequences and retain long-term information. Using Bi-LSTMs to process the CW radar data allows for the detection of heart and respiratory frequencies with high precision, showcasing an edge over traditional methods as well as the standalone CNN model discussed in the preceding section.

Figure 1 shows the architecture of the Bi-LSTM model proposed in this paper. It consists of a two-layer bidirectional LSTM with 128 units (layer A and layer D), a normalization layer (layer B), and a 1D global pooling layer (layer D) to reduce the dimensionality of the features extracted via the Bi-LSTM network. The 1D global pooling layer can help prevent overfitting and improve the generalization of the model. Additionally, it can save computational resources by reducing the number of parameters required for processing 1D sequential data. Our output layer is comprised of four units of fully connected layers, which corresponds to the desired outputs for the regression model. In the case of the classification model, the outputs correspond to the three physiological states of the driver, as shown in Figure 1.

Combining the benefits of temporal feature extraction and long-term memory retention, the proposed Bi-LSTM model offers a powerful solution for analyzing the vital signs measured via the CW radar.

### 2.4. CRNN

In the previous sections, we have seen the individual strengths of CNN and Bi-LSTM in detecting heart and respiratory frequencies from signals measured using the CW radar data. The CNN offers excellent spatial feature extraction capabilities, while the Bi-LSTM effectively handles long-term temporal dependencies within the vital sign sequences.

In this section, we present a CRNN architecture developed to detect heart rate and respiratory frequency from vital signs measured via the CW radar. This architecture combines the benefits of CNNs and Bi-LSTMs into a unified model, enabling precise and efficient extraction of spatial and temporal features.

Our CRNN architecture, illustrated in Figure 1, comprises a Conv-1D layer (layer A) of 128 units with a filter size of 512 for the initial extraction of features from CW radar signals. Following the phase of convolution, a bidirectional LSTM layer (layer C) of 128 units is used to comprehend the long-term temporal dependencies of these vital signs. This ability to effectively handle past and future information renders our model particularly suited to the sequential nature of heart rate and respiratory frequency data. The Bi-LSTM phase is followed by a Global Average Pooling layer (layer D) to reduce computational complexity while retaining the essence of key features. Subsequently, a series of dense layers are used and the final layer produces estimates of the heart rate and respiratory frequency for the regression output and it determines the physiological state of the driver for the classification output, as shown in Figure 1.

In conclusion, our CRNN architecture uses the local feature extraction capability of the CNN and the expertise over long-term temporal dependencies of the Bi-LSTM to provide a robust method for analyzing the vital signs measured via the CW radar.

## 3. Experience

### 3.1. Data

This section introduces the operating principle of the CW radar for vital signs’ measurements, specifically heart and respiratory rates. We will present two distinct databases used for training the Deep Learning models. The first database contains simulation data generated by MATLAB, while the second one contains real data measured via a 24 GHz CW radar.

#### 3.1.1. Simulation Data: Basic Principles of CW Radar Operation

The CW radar generates sinusoidal electromagnetic waves using a local oscillator (LO) [12,14]. These waves are then amplified via a power amplifier (PA). Mathematically, the transmitted signal, T(t), can be expressed as:

(1) $$T\left(t\right)=A_{T}\cos\left(2\pi ft+\phi \left(t\right)\right)$$

where *f* represents the frequency of the transmitted signal, ϕ(t) denotes the phase noise of the LO, and $A_{T}$ is the amplitude of the transmitted signal.

The transmitted waves reflect off of a moving object, such as a human body, and the reflected signal experiences a frequency shift due to the Doppler effect. The motion of the body comprises three components: respiration (xr), heartbeat (xc), and random body movements (xm). This gives rise to the received signal R(t), which can be mathematically expressed as:

(2) $$R\left(t\right)=A\cos\left[2\pi ft-\frac{4\pi d0}{\lambda }-\frac{4\pi x(t)}{\lambda }+\phi \left(t-\frac{2d0}{c}\right)\right]+N\left(t\right)$$

Here, *A* is the amplitude of the received signal, λ is the wavelength of the signal, *c* is the speed of light, d0 represents the initial distance between the CW radar and the body, x(t)=xr+xc+xm denotes the displacement of the human body surface, and N(t) is the signal noise.

(3) $$R\left(t\right)=A\cos\left[2\pi ft-\frac{4\pi d0}{\lambda }-\frac{4\pi xr(t)}{\lambda }-\frac{4\pi xc(t)}{\lambda }-\frac{4\pi xm(t)}{\lambda }+\phi \left(t-\frac{2d0}{c}\right)\right]+N\left(t\right)$$

**Figure 3:**
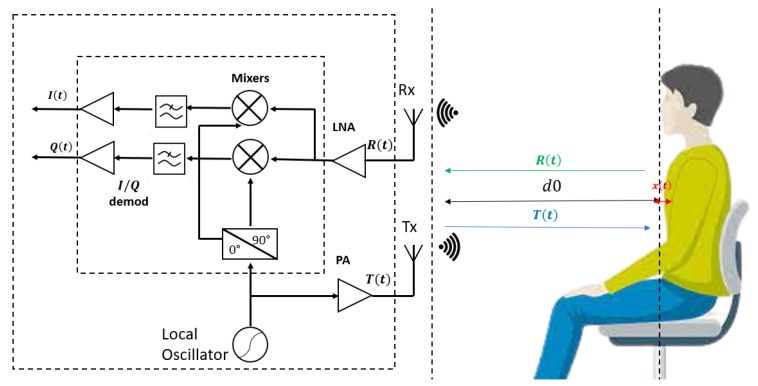
Fundamental mechanism of CW radar.

Upon reflection, the signal mixes with the local oscillator’s signal, producing two foundational signals: the in-phase signal I(t) and the quadrature-phase signal Q(t). This process is depicted in Figure 3, which illustrates the CW radar system and its associated components. The quadrature signal is shifted by 90° relative to the carrier. These signals can be expressed as:

(4) $$I\left(t\right)=A\cos\left[2\pi ft+\frac{4\pi d0}{\lambda }+\frac{4\pi xr(t)}{\lambda }+\frac{4\pi xc(t)}{\lambda }+\frac{4\pi xm(t)}{\lambda }+\Delta \phi \left(t\right)\right]+N_{I}\left(t\right)$$

(5) $$Q\left(t\right)=A\sin\left[2\pi ft+\frac{4\pi d0}{\lambda }+\frac{4\pi xr(t)}{\lambda }+\frac{4\pi xc(t)}{\lambda }+\frac{4\pi xm(t)}{\lambda }+\Delta \phi \left(t\right)\right]+N_{Q}\left(t\right)$$

In these equations, it is posited that the amplitudes of I(t) and Q(t) coincide [14], and $N_{I}\left(t\right)$ and $N_{Q}\left(t\right)$ represent the noise components for the in-phase and quadrature-phase signals, respectively, while Δϕ(t) represents any additional phase shift in the signal.

For these, the baseband signal, B(t), is derived as:

(6) B(t)=I(t)+jQ(t)

where *j* is the imaginary unit. For simplification, this signal is then expressed in exponential form using Euler’s formula:

(7) $$B\left(t\right)=A_{b}s\left(t\right)\exp\left[j\cos\left(2\pi Frt\right)\right]\exp\left[j\cos\left(2\pi Fct\right)\right]+N\left(t\right)$$

In this equation, $A_{b}=A\exp\left(j\frac{4\pi d0}{\lambda }\right)$, $s\left(t\right)=\exp[j\frac{4\pi xm(t)}{\lambda }+\Delta \phi \left(t\right)]$, and $N\left(t\right)=N_{i}\left(t\right)+jN_{q}\left(t\right)$.

#### 3.1.2. Simulation Data: Generation Procedure

We use a dataset generated by MATLAB for our research (simulation part), which serves to train and test our Deep Learning (DL) models of regression. This dataset comprises 3000 baseband signals from the CW radar, labeled with the heart rate, Fc, and the respiration rate, Fr, representing 30 subjects in a normal state, with each signal in Equation (7) having 5001 samples and a varying Signal to Noise Ratio (SNR). For our study, the CW radar is used to generate baseband signals, so we chose 30 heart and respiratory rate values within their normal ranges: Fc [0.83–2] Hz and Fr [0.16–0.33], and we also varied the SNR from −10 to 10 dB for each case. This is performed to illustrate the impact across 100 different environments. In this setup, the CW radar in Figure 3 and the individual are positioned 1 m apart as shown below Table 1. In addition, to estimate the performance of the regression models, we generated an additional dataset using MATLAB, containing 15 new values for the heart and respiratory rates to represent 15 subjects in different physiological states (fatigue or drowsiness, normal state, and stress). Each value of Fc and Fr represents an individual, and for each case, the SNR is varied between −10 and 10 dB to produce 100 signals with identical Fc and Fr in 100 distinct environments. We selected five values in the normal ranges (Fc [0.83–2] Hz, Fr [0.16–0.33]) to represent individuals in a normal state. It should be noted that any change in the physiological state results in modifications to the vital signs. For individuals displaying signs of fatigue or drowsiness, we chose five values below these normal ranges. Conversely, for those experiencing stress, we selected five values above the normal ranges. This distinction aims to capture the typical variations in heart and respiration rates associated with different physiological states. The reason why we generated two different databases is to test the ability of the different DL regression models proposed in the article to detect and extract the exact value of the heart rate and the respiratory rate for different physiological states of the driver, knowing that we only used a database that only represents the normal state of the driver. This is a strong point, particularly when validating our models with real measurement data (problem of lack of data [26]). The results of this test are presented in Section 4.

On the other hand, we have created a second database with 5300 labeled signals for our classification models. This time, we have selected 20 values for Fc and Fr to represent the driver’s normal state, 16 for fatigue and drowsiness, and 17 for stress. We varied the SNR for each value from −10 to 10 dB.

The normal range for an adult’s respiratory rate is between 10 and 20 breaths per minute, or 0.16 Hz and 0.33 Hz [29]. The accepted heart rate for adults is between 60 and 100 beats per minute, equivalent to 0.83 Hz and 1.67 Hz [30,31]. If a heart rate falls below 50 BPM (usually during sleep), it is referred to as bradycardia, while a rate over 100 BPM is called tachycardia. In terms of the amplitudes, the cardiac frequency amplitude (ac) ranges between 0.2 and 0.5 mm, and the respiratory frequency amplitude (ar) varies from 4 to 12 mm. The standard ranges for the values of ac and ar are ac [0.2–0.5] mm and ar [4–12] mm, respectively [32].

#### 3.1.3. Real Data

In this study, to both evaluate the performance of the DL models proposed and validate their accuracy on the real data, we used the clinical dataset provided in [33]. This dataset consists of 30 healthy subjects of different ages and sexes measured via the CW radar system based on Six-Port technology operating at 24 GHz in the ISM band. As a reference, they used an electrocardiogram measured simultaneously with the CW radar. The characteristics of the dataset are mentioned in [33]: Although this database was not created based on drivers, the signals it comprises will nevertheless allow us to test our regression and classification models on real signals.

To construct our dataset, we based it on the dataset proposed in [33], using the radar signals in phase and quadrature to construct the baseband signal for each subject (knowing that both signals are stored in mV). Each time, we obtain a signal representing a resting scenario of the person. This signal is of 1,215,200 samples with a sampling frequency of 2000 Hz. To keep the same principle of our simulation dataset, we have re-sampled the data with $f_{e}=100\;\mathrm{Hz}$ and we divided each signal into several signals of 5001 samples corresponding to 50.01 s as the acquisition time. To obtain the heart rate and respiratory rate values corresponding to each signal, several algorithms were used as follows. A normal FFT [34] was applied to the baseband signals measured via the CW radar to extract the respiratory rate and the results obtained were compared with the results of the cyclostationary algorithm [14]. For the heart rate, the R-peak algorithm [35] was applied to the EGG signals measured via the electrocardiogram.

We finally constructed a dataset to train and test our models, with 280 rows and 5003 columns, where each row corresponds to a signal of 5001 samples with two labels (heart rate and respiratory rate), that is, for the regression approach.

In addition, we have constructed a second dataset for validating our classification models. This second real dataset contained 612 label signals (219 representing resting (rst), 185 signals for Apnea (apn), and 208 representing Valsalva (vals) [33]). As the lack of data measured via the CW radar represents the different states of the driver (drowsiness or fatigue, normal state or resting, and stress), in order to evaluate our classification models, we used a dataset of the three scenarios mentioned above (Resting represents the normal state of the person, Apnea refers to a temporary pause in breathing, and finally, Valsalva represents a breathing technique involving a forceful exhalation against a closed airway, which can affect heart rate and blood pressure). The results of the regression and classification models are represented in Section 4.

### 3.2. Training, Test, and Evaluation Networks

In this context, all regression models were trained for 60 epochs with a batch size of 64. The Adam optimizer with a learning rate of 0.001 was used to minimize the Root Mean Squared Error (RMSE), which measures the difference between the model predictions and the actual data. Furthermore, the RMSE is used as an evaluation metric for assessing the performance in the regression output. As for the classification models, they have been trained for 60 epochs with a batch size of 64 and an Adam optimizer with a learning rate of 0.0001. The loss function chosen was the categorical cross-entropy, and the model performance was evaluated using the accuracy metric.

To evaluate the performance of our models, we devised our simulation dataset as follows: 64% for the training, 16% for the validation, and 20% for the test with a random state of 4. We will use the test dataset to estimate the accuracy and performance of each model (Bi-LSTM, 1D-CNN, CRNN, and TCN). In the case of the real dataset, we have the vital signs measured via the CW radar for 30 healthy subjects. We partitioned this dataset into three segments: 17 subjects for training, five for validation, and eight for testing. This partitioning pertains to the regression dataset which contains the signal representing the normal state of the person or the resting scenario. In addition, for the classification dataset, we have just an available dataset for 24 individuals representing the three scenarios of resting, Valsalva, and apnea. We devised our classification dataset as follows: 14 subjects for training, four for validation, and six for testing.

Several statistical indicators or static tests are used to evaluate the architectures of neural networks. In this article, three statistical indicators have been used for the regression models, namely the correlation coefficient R2_score, the root mean square error (RMSE), and the Mean Absolute Error (MAE) to quantify the accuracy of continuous predictions. Four other statistical indicators have been used to evaluate the classification models: accuracy, precision, recall, and the F1_score.

(8) $$RMSE=\sqrt{MSE}=\sqrt{\frac{1}{N}\sum {\left(y_{i}-Y_{pred}\right)}^{2}}$$

(9) $$R2\_score=1-\frac{\sum {\left(y_{i}-Y_{pred}\right)}^{2}}{\sum {\left(y_{i}-Y_{moy}\right)}^{2}}$$

(10) $$MAE=\frac{1}{N}\sum \left|y_{i}-Y_{pred}\right|$$

(11) $$Accuracy=\frac{All_{true}}{All}=\frac{TP+TN}{TP+TN+FP+FN}$$

(12) $$Precision=\frac{True_{Positives}}{Predicted_{Positives}}=\frac{TP}{TP+FP}$$

(13) $$Recall=\frac{True_{Positives}}{All_{ActualPositives}}=\frac{TP}{TP+FN}$$

(14) $$F1\_score=2\cdot \frac{Recall\cdot Precision}{Recall+Precision}$$

where:

$Y_{pred}$ is the value simulated by the model;

$y_{i}$ is the measured value;

$Y_{moy}$ is the mean of the measured values, and *N* is the number of samples.

TP (True Positives): The number of observations that were correctly classified as positive by the model.

TN (True Negatives): The number of observations that were correctly classified as negative by the model.

FP (False Positives): The number of observations that were incorrectly classified as positive. The model predicted the observation was positive when it was actually negative.

FN (False Negatives): The number of observations that were incorrectly classified as negative. The model predicted the observation was negative when it was actually positive.

To evaluate the performance of each regression model, we used an adapted version of R2score, referred to as the R2score* indicator, which can be mathematically expressed as:

(15) $$R2\mathrm{score}*=1-\frac{\sum {\left(y_{i}-Y_{pred}\right)}^{2}}{\sum (y_{i}^{2})}$$

The reason for using R2score* instead of R2score is the use of different datasets, each containing 100 signals with varying snr [−10, 10] and the same *y* (Fc and Fr). The results obtained were presented in Section 4.3.

## 4. Results and Discussion

In this section, we will present a comparison of the Models’ parameters, and after that, we will present both the results obtained using the simulation data in Section 4.2 and the results of real or measured data in Section 4.3.

### 4.1. Comparison of Models’ Parameters

Table 2 and Table 3 provide a comprehensive and detailed overview of the complexity of the four neural network models presented in this paper: CNN, TCN, Bi-LSTM, and CRNN. We observe that in both classification and regression tasks, CNN models display the largest number of total and trainable parameters, about 18.7 million, reflecting a higher complexity of this model compared to the TCN, Bi-LSTM, and CRNN models.

The TCN, Bi-LSTM, and CRNN models have significantly fewer total and trainable parameters, with the CRNN model having the lowest number when applied to regression tasks, despite being tasked with predicting two outputs (heart and respiratory rates) as well as the three outputs (fatigue, normal state, and stress) in the classification tasks.

### 4.2. Simulation Data

**Figure 4:**
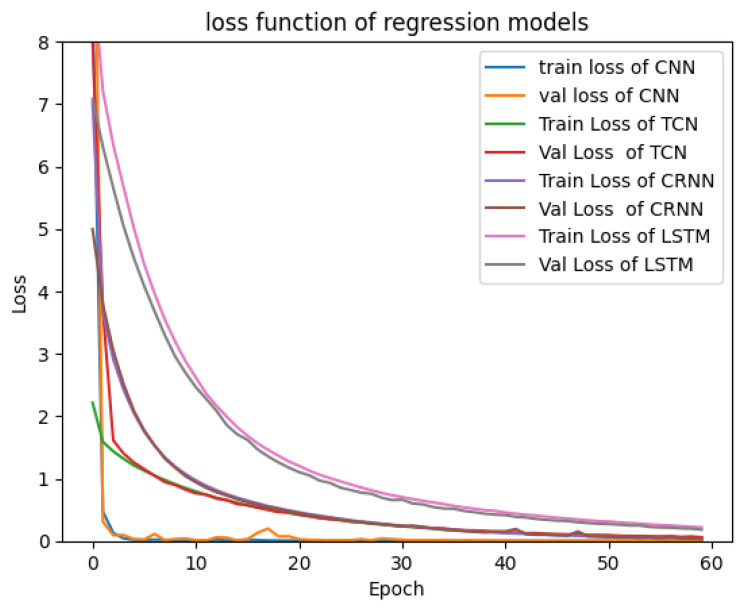
Loss function of regression models.

**Figure 5:**
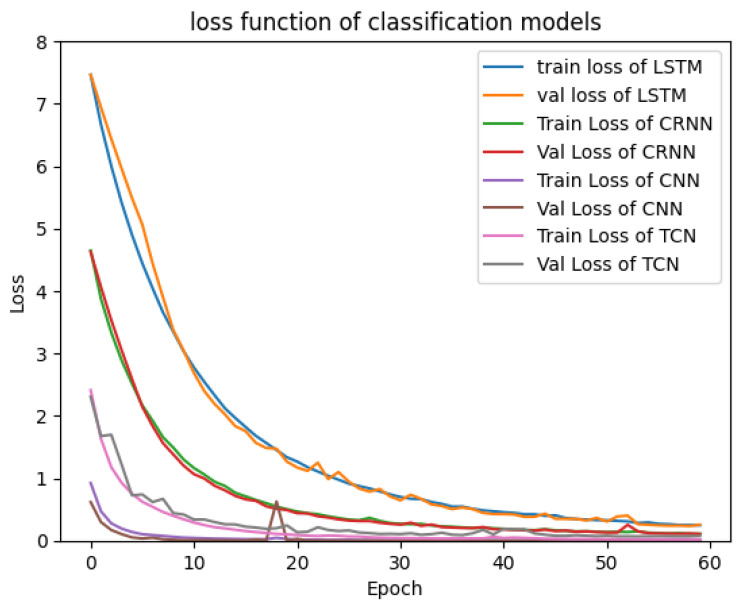
Loss function of classification models.

This subsection presents the results of various tests carried out using simulation databases for the different models presented in Section 2. Figure 4 and Figure 5 show the validation and training loss curves of the regression and classification models with a simulation dataset of 3000 labeled signals of the heart rate and respiration rate in normal ranges for the regression output and a simulation dataset labeled with three classes representing the different physiological states of the driver (drowsiness, normal state, and stress) for the classification outputs. The training loss refers to the model’s error and it shows the learning ability of each model in each epoch. The validation loss shows the model’s ability to recall or generalize to new validation data. We observe that the loss functions of each model progressively converge to a value close to 0.0, demonstrating that the models have a high ability and capability to learn from their training data. We observe that the 1D-CNN converges rapidly (in the 10 first epochs), as well as the TCN, Bi-LSTM, and CRNN models, which converge in around 30 epochs. Furthermore, the various curves dedicated to the validation data confirm that there is no overfitting for all proposed models.

**Figure 6:**
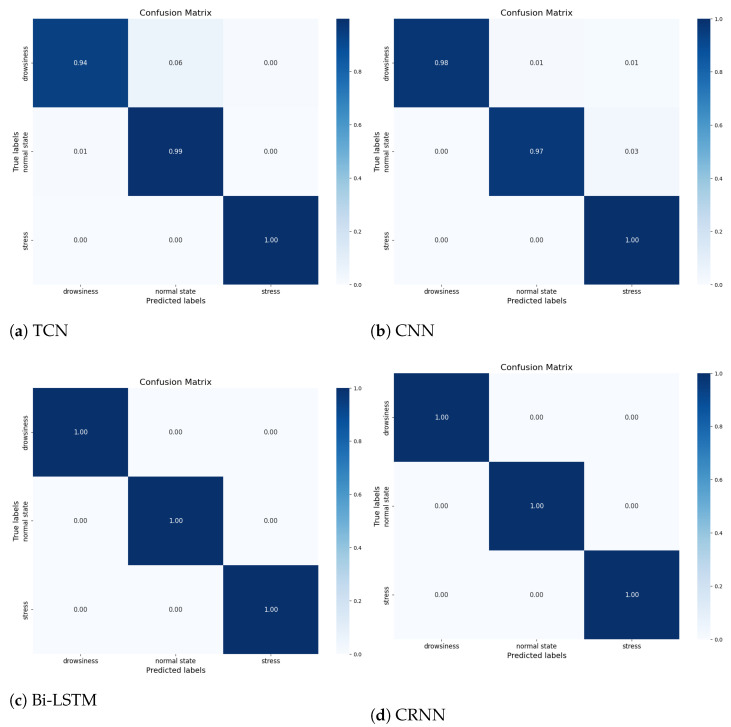
Confusion Matrix for Each Model Using the Simulated Dataset, Dependent on Individual variances.

Table 4 represents the values of the three static indicators RMSE, MAE, and R2-score for the different regression models tested to resolve our problems in estimating the heart and respiration rates. These indicators were calculated after the accuracy values were obtained after ten test compilations for each model, in order to assess their accuracy. The table shows that the CRNN and Bi-LSTM models perform better than TCN and CNN in detecting heart and respiratory rates (Bi-LSTM indicators values: RMSE_Fc=0.134, $RMSE\_Fr=2.5\times 10^{-2}$, $MAE\_Fc=1.3\times 10^{-2}$, $MAE\_Fr=5\times 10^{-2}$, R2_Fc=93.8%, and R2_Fr=93.1%) from the temporal signals at the level of both indicators. These results are due to the ability of the Bi-LSTM model to process temporal sequences and to keep the information in the long term, as opposed to the CNN ($RMSE\_Fc=6.8\times 10^{-2}$, $RMSE\_Fr=2.3\times 10^{-2}$, $MAE\_Fc=5.9\times 10^{-3}$, $MAE\_Fr=3.3\times 10^{-3}$, R2_Fc=93.2%, and R2_Fr=88.7%). Table 5 represents the values of different indicators evaluating our classification models, and Figure 6 represents the confusion matrix of each model.

The accuracy of the four classification models in identifying and classifying the physiological state of the driver based on the vital signs measured via the CW radar is demonstrated in Figure 5 and Table 5. Figure 6 represents the confusion matrix of the different models proposed in our article. It is a valuable tool for evaluating the performance of the classification between the four models. The confusion matrix of the CNN, TCN, Bi-LSTM, and CRNN models has no difficulty in identifying and classifying the three states of the driver.

Table 6 above illustrates the accuracy (R2score*) of each model in predicting the exact values of the heartbeat and breathing rates of each state (drowsiness, normal state, and stress) when we tested our model with the additional dataset (Section 3).

**Figure 7:**
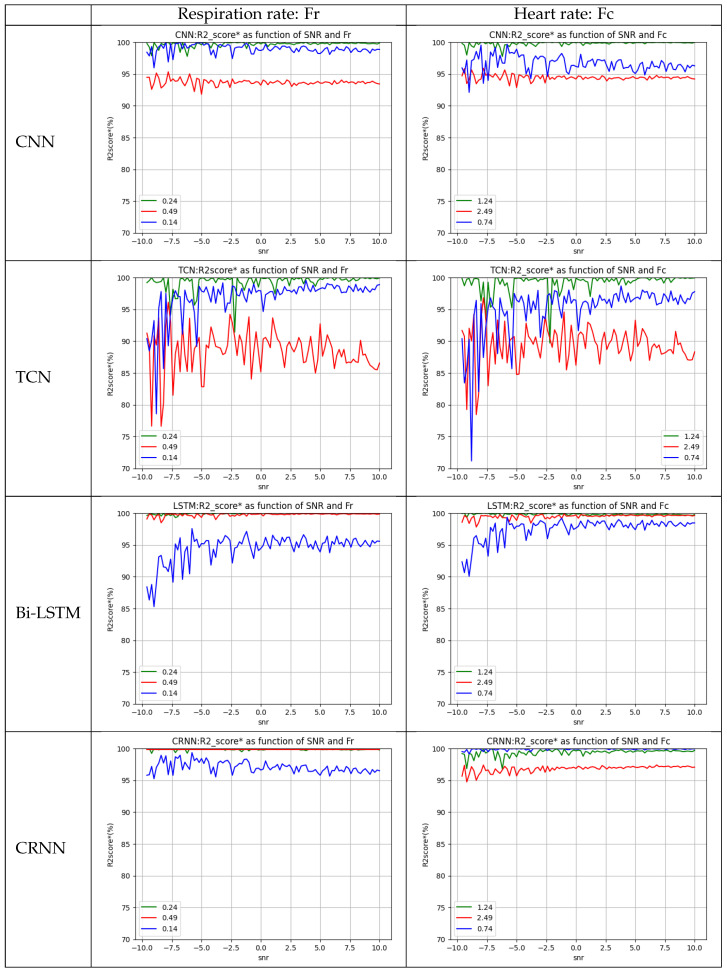
Comparative accuracy curves: predicting heart and respiration rates based on physiological state.

Figure 7 represents the accuracy of each model in predicting the exact value of the heart and respiration rate in every state regardless of the SNR value (the blue curve represents drowsiness or fatigue, the green curve represents the normal state, and the red curve represent the stress). These results show us that we can also estimate the value of the heart rate and breathing rate independently of the person.

Following the preceding graphs, it is observed that the CNN, TCN, Bi-LSTM, and CRNN models proposed can predict the values of respiratory frequency: Fr with a robust precision (greater than 80%) regardless of SNR and the physiological state of the person. On the other hand, heart rate values within the normal range and values representing fatigue and drowsiness can be accurately predicted regardless of the SNR value, but the heart rate of a stress state cannot be accurately predicted.

**Figure 8:**
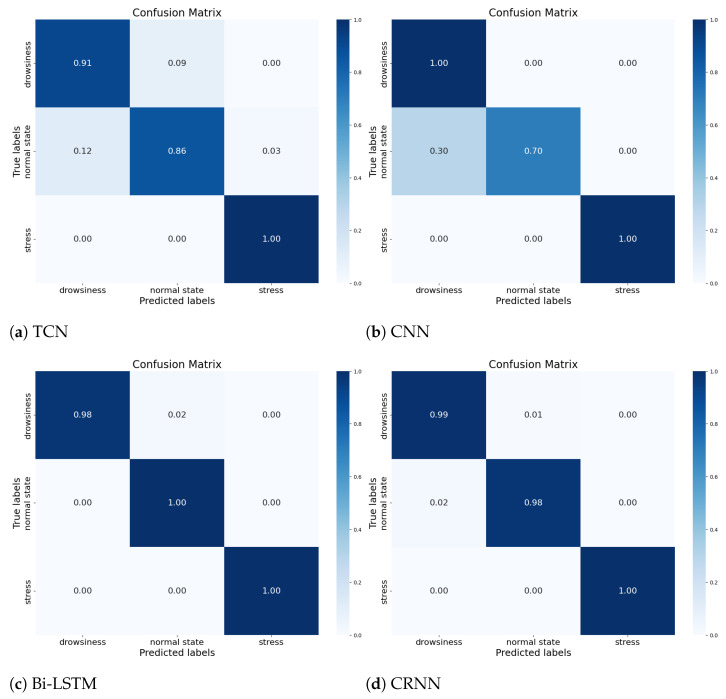
Confusion Matrix for Each Model Using the Simulated Dataset, Independent of Individual Variances.

The confusion matrices, in Figure 8 depicted below, show the results achieved by our classification models, using the same database as those referenced in Figure 7 and Table 6. From the displayed results, we note that during the simulation phase, thanks to our models, we are able to estimate the heart and respiratory rates, as well as classify the physiological state of the driver independent of the SNR and individual variances, provided that the person is in good health.

### 4.3. Real Data

**Figure 9:**
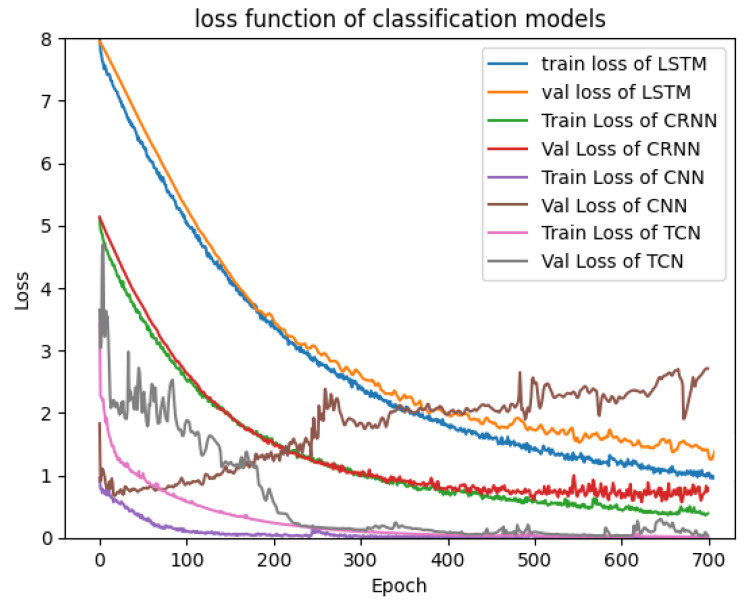
Loss function of classification models.

**Figure 10:**
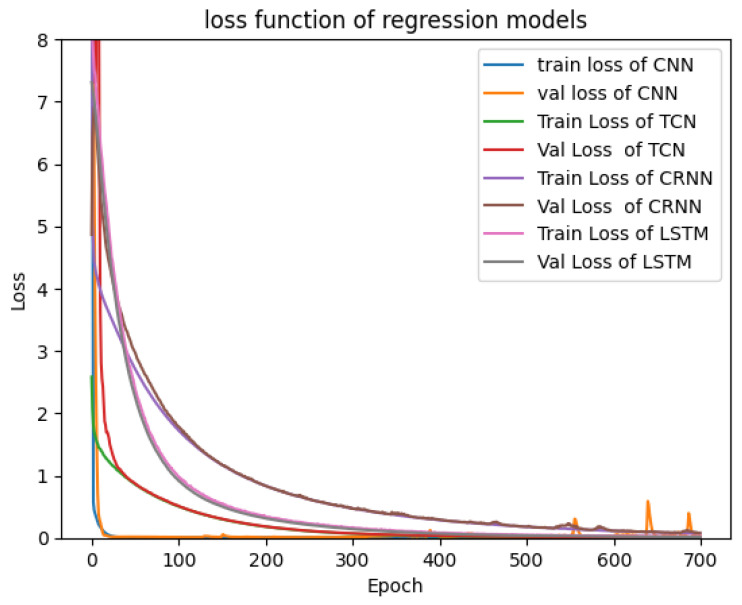
Loss function of regression models.

This subsection presents the results obtained by the use of real or measured databases for the different models presented in Section 2. Figure 9 and Figure 10 show the validation and training loss curves of the regression and classification models with the real data presented in Section 3, in which the regression database containing 260 signals represents 28 persons in a normal state and the labeled heart and respiration rate in normal ranges. The classification dataset contains 170 signals representing different states of persons (Valsalva, apnea, and resting). The different curves of our models demonstrate the ability of each model to make a relation between the input (vital signs) and the output (classification of the state or the prediction of the heart and preparation values). We observe that in Figure 9 and Figure 10, all curves converge progressively to 0.0, but with more epochs than for the simulating dataset, with the exception of the val loss curve of the CNN classification model, which diverges. This shows that the CNN is too complex in the case of the classification task and realizes an overfitting. This is not the case for the regression task. However, our challenge is to maintain the same architecture used for the simulation data and to test its performance on the real data.

Table 7 presents the values of the static indicators RMSE and MAE for the different proposed regression models (CNN-1D, TCN, Bi-LSTM, and CRNN) with a window size of 50 s, tested with the real data [33] in order to validate the architecture of our four proposed regression models in predicting heart and respiration rates. It is essential to note the intervals within which these predictions are being made. For the respiratory frequency (Fr), the interval lies between 0.16 and 0.33 Hz, while for the cardiac frequency (Fc), it is in the range of 0.83–2 Hz. Given these narrow intervals, even minor prediction errors can be significant. From the values shown in Table 7, it is clear that all models perform better in predicting the respiratory rate (Fr) than the heart rate (Fc). Specifically, the Bi-LSTM model outperforms the others, demonstrating the lowest RMSE and MAE for both Fr and Fc. For Fr, the Bi-LSTM and TCN models exhibit relatively low RMSE and MAE errors, with values as low as 0.057 and 0.053, respectively. Conversely, for the prediction of heart rate Fc, the Bi-LSTM and CRNN models yield lower RMSE and MAE error values, ranging between 0.11 and 0.147. Notably, the MAE values obtained for each of our models are significantly lower compared to the MAE values reported in another study [27,36]. The study [27] used a UWB radar for measurement and employed Convolutional Neural Networks with a window size of 15 s for analysis. Furthermore, the study in [36], which employed the same dataset as ours from [33] and used a Standard LSRM for extracting the heart rate (Fc) with a window size of 20 s, will be discussed in Table 7 below.

**Figure 11:**
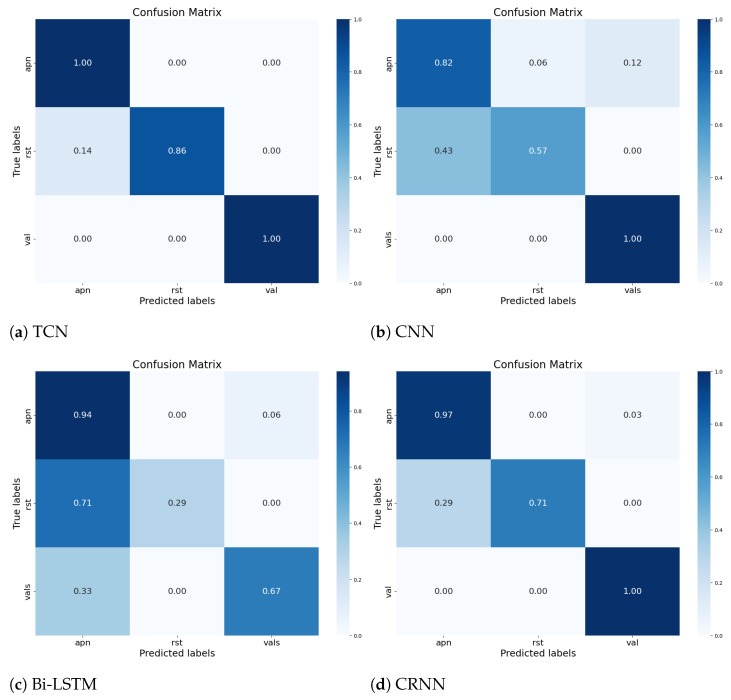
Confusion matrix for each model Using the Real Dataset, Independent of Individual Variances.

By examining the confusion matrices for each model in Figure 11, we can see where each model struggles. For example, the Bi-LSTM model has trouble distinguishing classes resting (rst) and Valsalva (val) from class apnea (apn), with only a 29% and 67% accuracy, respectively, for these classes. Similarly, the 1D-CNN model struggles to distinguish class rst from class apn, with an accuracy of only 57% for class rst. This is unsurprising given the architecture’s lack of generalization during learning (overfitting). Lastly, the confusion matrices for the CRNN and TCN models indicate that these models perform relatively well for all classes, although the CRNN model has a slightly lower performance for class rst. these results are consistent with the performance of each model in Table 7. That shows the disability of 1D-CNN and Bi-LSTM to learn their input raw signal in the short term and in the long term, respectively. However, for CRNN, the Bi-LSTM layer is able to learn the series extracted via the Conv-1D layer.

Table 8 shows an evaluation of the performance of our four classification models, 1D-CNN, TCN, Bi-LSTM, and CRNN, using different metrics of evaluation. Of these models, the TCN has the highest performance across all metrics, with an accuracy, precision, recall, and f1 score of 97%. The CRNN follows closely, with a performance exceeding 93% on all metrics. However, 1D-CNN and Bi-LSTM have relatively lower performance with scores for accuracy, precision, recall, and F1_score all below 85%. It is notable that the authors in [37,38] employed the same dataset we used from [37] to train and validate their deep learning classification models. In [29], the research introduced an ANN model to classify five physiological states: Resting, sleep apnea, Valsalva, tilt up, and tilt down, achieving an accuracy of 83%. Meanwhile, in [38], the study proposed a combination of CNN and GRU to classify four scenarios: Resting, sleep apnea, Valsalva, and tilt, reporting an accuracy of 95.52%, a precision of 96.35%, a recall of 94.74%, and an F1_score of 95.40%.

## 5. Conclusions

This paper presents a non-contactless approach for detecting and extracting heart and respiration rates to monitor the driver’s physiological state. This approach is based on the concatenation of the measurement of the vital signs via a 24 GHz CW radar and the analysis via different Deep Learning models based on temporal neural networks. The goal is to estimate the heart and respiration rates and to classify the physiological state of the driver. All proposed solutions have shown a robust performance using the simulation data for detecting heart and respiration rate values simultaneously in the normal range and also for accurately classifying the different physiological states of the driver, regardless of the value of the SNR. The CRNN was particularly noteworthy as the best performer among them. As part of our future work, we plan to test our Deep Learning models using real data representing different states of the driver such as drowsiness, normal state, and stress. Furthermore, special attention will be given to the propagation channel, taking into account body movements and noise. We also aim to refine our models to be able to detect with high precision the values of heart rate and respiratory rate, not only in the normal range, but also in a different physiological state of the driver.

## Figures and Tables

**Table 1:** Simulation parameters.

Parameter	Value
Radar Frequency	24 GHz
Fc	[0.83–2] Hz
Fr	[0.16–0.33] Hz
ac	[0.2–0.5] mm
ar	[4–12] mm
Distance	[0.6–1] m

**Table 2:** Comparison of classification model parameters.

	CNN	TCN	Bi-LSTM	CRNN
Total params.	18,697,379	559,907	547,747	348,195
Trainable params.	18,697,251	556,835	547,107	348,067
Non-trainable params.	128	3072	640	128

**Table 3:** Comparison of regression model parameters.

	CNN	TCN	Bi-LSTM	CRNN
Total params.	18,697,362	559,890	547,730	348,178
Trainable params.	18,697,234	556,818	547,090	348,050
Non-trainable params.	128	3072	640	128

**Table 4:** Average R2 scores of RMSE and MAE for each regression model after 10 tests using the simulated Dataset.

	1D-CNN	Bi-LSTM	CRNN	TCN
**RMSE**				
Fr				
Average	$2.3\times 10^{-2}$	$2.5\times 10^{-2}$	$5\times 10^{-2}$	$4.2\times 10^{-2}$
Standard Deviation	$1.2\times 10^{-2}$	$1.1\times 10^{-4}$	$1.7\times 10^{-2}$	$1.8\times 10^{-4}$
Fc				
Average	$6.8\times 10^{-2}$	0.134	$5.8\times 10^{-2}$	0.232
Standard Deviation	$3.3\times 10^{-2}$	$3.5\times 10^{-4}$	$3.5\times 10^{-2}$	$1.1\times 10^{-2}$
**MAE**				
Fr				
Average	$5.9\times 10^{-3}$	$1.3\times 10^{-2}$	$1.6\times 10^{-2}$	$2.5\times 10^{-2}$
Standard Deviation	$4.5\times 10^{-3}$	$1.1\times 10^{-2}$	$1.2\times 10^{-2}$	$2.7\times 10^{-2}$
Fc				
Average	$3.3\times 10^{-2}$	$5\times 10^{-2}$	$5.8\times 10^{-2}$	$5\times 10^{-2}$
Standard Deviation	$2.3\times 10^{-2}$	$4\times 10^{-2}$	$3.5\times 10^{-2}$	$4\times 10^{-2}$
**R2 Score**				
Fr				
Average (%)	88.7	93.1	87.7	76.9
Standard Deviation	$9.7\times 10^{-2}$	$4.4\times 10^{-2}$	0.123	$4.3\times 10^{-2}$
Fc				
Average (%)	93.2	93.8	97.23	74.91
Standard Deviation	0.207	$3.5\times 10^{-2}$	$2.5\times 10^{-4}$	$5.4\times 10^{-2}$

**Table 5:** Evaluation of different classification models using the simulated Dataset.

	1D-CNN	TCN	Bi-LSTM	CRNN
Accuracy	97.9%	98.2%	99%	99%
Precision	98%	98.2%	99%	99%
Recall	97.9%	98.2%	99%	99%
F1_score	97.9%	98.1%	99%	99%

**Table 6:** Average R2score* (%) for Each Regression Model to predict heart and respiration rates in different physiological states.

	Model	Fr	Fc
**CNN**	Drowsiness	95.7	94.3
	Normal state	97	98
	Stress	88	90
**Bi-LSTM**	Drowsiness	92.5	96
	Normal state	98	98
	Stress	96	90
**CRNN**	Drowsiness	95	95
	Normal state	98	98
	Stress	90	94
**TCN**	Drowsiness	90	82.5
	Normal state	97	96
	Stress	88	85

**Table 7:** Performance metrics of regression models for predicting respiratory and cardiac frequencies Using The Real Dataset.

	1D-CNN	Bi-LSTM	CRNN	TCN	[27]	[36]
**RMSE**						
Fr						
Average	0.136	0.057	0.082	0.058	-	-
Standard Deviation	0.078	0.024	0.042	0.024	-	-
Fc						
Average	0.234	0.142	0.2	0.155	-	2.35
Standard Deviation	0.132	0.091	0.129	0.095	-	-
**MAE**						
Fr						
Average	0.111	0.043	0.07	0.053	2.67	-
Standard Deviation	0.078	0.024	0.042	0.024	1.45	-
Fc						
Average	0.192	0.11	0.147	0.119	4.78	-
Standard Deviation	0.132	0.091	0.135	0.095	0.80	-

**Table 8:** Evaluation of different classification models Using the Real Dataset.

	1D-CNN	TCN	Bi-LSTM	CRNN	[37]	[38]
Accuracy	79.54%	97.72%	81.81%	93.18%	83%	95.52%
Precision	83.32%	97.79%	84.38%	93.87%	-	96.35%
Recall	79.54%	97.72%	81.81%	93.18%	-	94.74%
F1_score	80.45%	97.65%	79.65%	93.01%	-	95.40%

## Data Availability

We utilized existing data from another paper, which is cited in the references.

## Abbreviations

The following abbreviations are used in this manuscript:

Fc	Heart rate
Fr	Respiration rate
CW	Continuous Wave
SNR	Signal Noise Ratio
FFT	Fast Fourier Transform
1D-CNN	Convolutional Neural Network (One-dimensional)
Bi-LSTM	Bidirectional Long Short-Term Memory
RNN	Recurrent Neural Network
CRNN	Convolutional Recurrent Neural Network
ANN	Artificial Neural Network
TCN	Temporal Convolutional Network
EMD	Empirical Mode Decomposition
UWB	Ultra wideband
MAE	Mean absolute error
RMSE	Root Mean Square Error
PPG	Photoplethysmography
ECG	Electrocardiography
CWT	Continuous Wavelet Transform
ISM	Industrial, Scientific, and Medical band
GRU	Gated Recurrent Unit
WT	Wavelet Transform

## References

[1] Li G., Chung W.Y. (2022). Electroencephalogram-Based Approaches for Driver Drowsiness Detection and Management: A Review. Sensors.

[2] Wang H., Dragomir A., Abbasi N.I., Li J., Thakor N.V., Bezerianos A. (2018). A Novel Real-Time Driving Fatigue Detection System Based on Wireless Dry EEG. Cogn. Neurodyn..

[3] Arefnezhad S., Hamet J., Eichberger A., Frühwirth M., Ischebeck A., Koglbauer I.V., Moser M., Yousefi A. (2022). Driver Drowsiness Estimation Using EEG Signals with a Dynamical Encoder–Decoder Modeling Framework. Sci. Rep..

[4] Ganapathy N., Baumgärtel D., Deserno T.M. (2021). Automatic Detection of Atrial Fibrillation in ECG Using Co-Occurrence Patterns of Dynamic Symbol Assignment and Machine Learning. Sensors.

[5] Clark N., Sandor E., Walden C., Ahn I.S., Lu Y. A Wearable ECG Monitoring System for Real-Time Arrhythmia Detection. Proceedings of the 2018 IEEE 61st International Midwest Symposium on Circuits and Systems (MWSCAS).

[6] Nazarian S., Lam K., Darzi A., Ashrafian H. (2021). Diagnostic Accuracy of Smartwatches for the Detection of Cardiac Arrhythmia: Systematic Review and Meta-Analysis. J. Med. Internet Res..

[7] Romano C., Schena E., Silvestri S., Massaroni C. (2021). Non-Contact Respiratory Monitoring Using an RGB Camera for Real-World Applications. Sensors.

[8] Huang P.-W., Wu B.-J., Wu B.-F. (2021). A Heart Rate Monitoring Framework for Real-World Drivers Using Remote Photoplethysmography. IEEE J. Biomed. Health Inform..

[9] Wusk G., Gabler H. (2018). Non-Invasive Detection of Respiration and Heart Rate with a Vehicle Seat Sensor. Sensors.

[10] Zhang Z., Nian Y., Chen J., He M. An Experimental Study to Optimize the Stepped-Frequency Continuous-Wave Radar Parameters for Noncontact Multi-Target Vital Sign Monitoring. Proceedings of the 2019 IEEE International Conference on Computational Electromagnetics (ICCEM).

[11] Seflek I., Acar Y.E., Yaldiz E. (2020). Small Motion Detection and Non-Contact Vital Signs Monitoring with Continuous Wave Doppler Radars. Elektron. Elektrotechnika.

[12] Malešević N., Petrović V., Belić M., Antfolk C., Mihajlović V., Janković M. (2020). Contactless Real-Time Heartbeat Detection via 24 GHz Continuous-Wave Doppler Radar Using Artificial Neural Networks. Sensors.

[13] Kazemi S., Ghorbani A., Amindavar H., Li C. (2013). Cyclostationary Approach for Heart and Respiration Rates Monitoring with Body Movement Cancellation Using Radar Doppler System. arXiv.

[14] Sekak F., Zerhouni K., Elbahhar F., Haddad M., Loyez C., Haddadi K. (2020). Cyclostationary-Based Vital Signs Detection Using Microwave Radar at 2.5 GHz. Sensors.

[15] Tu J., Lin J. (2016). Fast Acquisition of Heart Rate in Noncontact Vital Sign Radar Measurement Using Time-Window-Variation Technique. IEEE Trans. Instrum. Meas..

[16] Li M., Lin J. (2018). Wavelet-Transform-Based Data-Length-Variation Technique for Fast Heart Rate Detection Using 5.8-GHz CW Doppler Radar. IEEE Trans. Microw. Theory Technol..

[17] Petrovic V.L., Jankovic M.M., Lupsic A.V., Mihajlovic V.R., P-Bozovic J.S. (2019). High-Accuracy Real-Time Monitoring of Heart Rate Variability Using 24 GHz Continuous-Wavedoppler Radar. IEEE Access.

[18] Hernandez-Aguila M., Olvera-Cervantes J.L., Perez-Ramos A.E. (2022). et al. Methodology for the Determination of Human Respiration Rate by Using Doppler Radar and Empirical Modal Decomposition. Sci. Rep..

[19] Hu X., Jin T. (2016). Short-Range Vital Signs Sensing Based on EEMD and CWT Using IR-UWB Radar. Sensors.

[20] Saluja J., Casanova J., Lin J. (2020). A Supervised Machine Learning Algorithm for Heart-Rate Detection Using Doppler Motion-Sensing Radar. IEEE J. Electromagn. RF Microwaves Med. Biol..

[21] Arsalan M., Santra A., Will C. (2020). Improved Contactless Heartbeat Estimation in FMCW Radar via Kalman Filter Tracking. IEEE Sens. Lett..

[22] Khan F., Cho S.H. (2017). A Detailed Algorithm for Vital Sign Monitoring of a Stationary/Non-Stationary Human through IR-UWB Radar. Sensors.

[23] Wu Q., Mei Z., Lai Z., Li D., Zhao D. (2021). A Non-Contact Vital Signs Detection in a Multi-Channel 77 GHz LFMCW Radar System. IEEE Access.

[24] Iyer S., Zhao L., Mohan M.P., Jimeno J., Siyal M.Y., Alphones A., Karim M.F. (2022). mm-Wave Radar-Based Vital Signs Monitoring and Arrhythmia Detection Using Machine Learning. Sensors.

[25] Ye C., Toyoda K., Ohtsuki T. (2019). Blind Source Separation on Non-Contact Heartbeat Detection by Non-Negative Matrix Factorization Algorithms. IEEE Trans. Biomed. Eng..

[26] Wu S., Sakamoto T., Oishi K., Sato T., Inoue K., Fukuda T., Mizutani K., Sakai H. (2019). Person-Specific Heart Rate Estimation with Ultra-Wideband Radar Using Convolutional Neural Networks. IEEE Access.

[27] Choi S.H., Yoon H. (2023). Convolutional Neural Networks for the Real-Time Monitoring of Vital Signs Based on Impulse Radio Ultrawide-Band Radar during Sleep. Sensors.

[28] Bai S., Kolter J.Z., Koltun V. (2018). An Empirical Evaluation of Generic Convolutional and Recurrent Networks for Sequence Modeling. arXiv.

[29] Cretikos M.A., Bellomo R., Hillman K., Chen J., Finfer S., Flabouris A. (2008). Respiratory rate: The neglected vital sign. Med. J. Aust..

[30] American Heart Association All About Heart Rate (Pulse). American Heart Association, 2015. https://www.heart.org/en/health-topics/high-blood-pressure/the-facts-about-high-blood-pressure/all-about-heart-rate-pulse.

[31] Mayo Clinic Tachycardia. Mayo Clinic, 2021. https://www.mayoclinic.org/diseases-conditions/tachycardia/symptoms-causes/syc-20355127.

[32] Obeid D., Apostolidis A., Noun L., Challita E., Jrad N., Elhawary H. (2010). Multitunable microwave system for touchless heartbeat detection and heart rate variability extraction. Microw. Opt. Technol. Lett..

[33] Schellenberger S., Shi K., Steigleder T., Malessa A., Michler F., Hameyer L., Neumann N., Lurz F., Weigel R., Ostgathe C. (2020). A Dataset of Clinically Recorded Radar Vital Signs with Synchronised Reference Sensor Signals. Sci. Data.

[34] Ren L., Wang H., Naishadham K., Kilic O., Fathy A.E. (2016). Phase-Based Methods for Heart Rate Detection Using UWB impulse Doppler radar. IEEE Trans. Microw. Theory Tech..

[35] Sadhukhan E., Mitra M. (2012). R-Peak Detection Algorithm for ECG Using Double Difference and RR Interval Processing. Procedia Technol..

[36] Han-Trong T., Nguyen Viet H. (2022). An Efficient Heart Rate Measurement System Using Medical Radar and LSTM Neural Network. J. Electr. Comput. Eng..

[37] Slapničar G., Wang W., Luštrek M. (2021). Classification of Hemodynamics Scenarios from a Public Radar Dataset Using a Deep Learning Approach. Sensors.

[38] Özkaya U. (2023). Radar Vital Signs Detection by Using Optimized CNN + GRU Model. Res. Sq..

